# Biosynthesis, Characterization and Antibacterial Application of Novel Silver Nanoparticles against Drug Resistant Pathogenic *Klebsiella pneumoniae* and *Salmonella* Enteritidis

**DOI:** 10.3390/molecules26195996

**Published:** 2021-10-02

**Authors:** Md. Amdadul Huq, Shahina Akter

**Affiliations:** 1 Department of Food and Nutrition, College of Biotechnology and Natural Resource, Chung-Ang University, Anseong 17546, Korea; 2 Department of Food Science and Biotechnology, Gachon University, Seongnam 461-701, Korea

**Keywords:** *Massilia* sp. MAHUQ-52, biosynthesis, AgNPs, *K. pneumoniae*, *S.* Enteritidis

## Abstract

The present study highlights the biosynthesis of silver nanoparticles (AgNPs) using culture supernatant of *Massilia* sp. MAHUQ-52 as well as the antimicrobial application of synthesized AgNPs against multi-drug resistant pathogenic *Klebsiella pneumoniae* and *Salmonella* Enteritidis. Well-defined AgNPs formation occurred from the reaction mixture of cell-free supernatant and silver nitrate (AgNO_3_) solution within 48 h of incubation. UV-visible spectroscopy analysis showed a strong peak at 435 nm, which corresponds to the surface plasmon resonance of AgNPs. The synthesized AgNPs were characterized by FE-TEM, EDX, XRD, DLS and FT-IR. From FE-TEM analysis, it was found that most of the particles were spherical shape, and the size of synthesized nanoparticles (NPs) was 15–55 nm. EDX spectrum revealed a strong silver signal at 3 keV. XRD analysis determined the crystalline, pure, face-centered cubic AgNPs. FT-IR analysis identified various functional molecules that may be involved with the synthesis and stabilization of AgNPs. The antimicrobial activity of *Massilia* sp. MAHUQ-52 mediated synthesized AgNPs was determined using the disk diffusion method against *K. pneumoniae* and *S.* Enteritidis. Biosynthesized AgNPs showed strong antimicrobial activity against both *K. pneumoniae* and *S.* Enteritidis. The MICs of synthesized AgNPs against *K. pneumoniae* and *S.* Enteritidis were 12.5 and 25.0 μg/mL, respectively. The MBC of biosynthesized AgNPs against both pathogens was 50.0 μg/mL. From FE-SEM analysis, it was found that the AgNPs-treated cells showed morphological changes with irregular and damaged cell walls that culminated in cell death.

## 1. Introduction

Various metal nanoparticles (NPs) are synthesized due to their unique properties, such as electrical, optical, biological, catalytic, and magnetic characteristics and low-cytotoxicity and enhanced permeability [1,2,3]. Among different metal NPs, silver nanoparticles (AgNPs) have drawn countless attention due to their various applications in different sectors as cancer therapeutics, biosensors, antibiotics, anti-inflammatory and drug delivery, etc. [4,5,6,7,8,9,10,11]. Many recent studies reported that biogenic AgNPs exhibited strong antibacterial activity against different pathogenic microorganisms [9,12,13]. Studies also suggest that biosynthesized AgNPs have excellent anti-inflammatory and anti-cancer activities [5,6]. According to Fouda et al. [7,8], biogenic AgNPs showed strong activity in degrading various toxic chemicals. Recently, biosynthesized AgNPs have been used for food preservation, sanitization, water filtration, nanoinsecticides, nanopesticides and cosmetics, etc. [10,14,15]. Different physical and chemical methods have been applied for the synthesis of metal NPs. However, globally, it is realized that biological synthesis methods are safe, facile, and non-toxic compared to the expensive, toxic and dangerous chemical and physical processes [7,16]. Various destructive impacts of chemical and physical methods can be solved through the biosynthesis of NPs using different biological entities. Therefore, it is essential to develop a simple, cost-effective, non-toxic, and environmentally friendly approach for the facile and mass production of metal NPs using biological systems. Biological synthesis can be performed using either bacteria [17], fungi [18] or plant extracts [19,20]. Among various biological entities, bacterial-mediated synthesis of NPs is mostly preferred due to their largescale production, high growth rate and ease of handling [13,21]. Different studies showed the biosynthesis of AgNPs using bacteria including *Microvirga rosea, Paenibacillus anseongensis, Novosphingobium* sp., *Sporosarcina koreensis* DC4, *Terrabacter humi*, *Pseudomonas* sp., etc. [22,23,24,25,26,27]. Rhizosphere bacteria are considered to be useful in various sectors, such as in phytoremediation, biotransformation and biosynthesis of valuable products, to increase plant growth and productivity, etc. [22,28].

The emergence of multidrug-resistant (MDR) microorganisms due to the uncontrolled, immoderate and multiple uses of antibiotics and chemotherapeutics is a serious threat to the world population. *Klebsiella pneumoniae* is a Gram-negative, non-motile, facultative anaerobic, encapsulated, rod-shaped bacterium that can cause various types of healthcare-associated diseases, including pneumonia, wound or surgical site infections, bloodstream infections, and meningitis [29]. *Salmonella* Enteritidis is a food-borne pathogen and can cause various food-borne illnesses in humans including gastroenteritis when contaminated food is consumed. Salmonellosis is a common food-borne disease that is caused by the infection of *Salmonella*. *Salmonella* outbreaks are severe for those vulnerable people who are immunocompromised, young and old [30]. Recently, both *K. pneumoniae* and *S.* Enteritidis have shown resistance against different antibiotics [29,30]. The development of a new antimicrobial agent is the decisive solution for this issue. Therefore, biosynthesized AgNPs may be a promising agent to control these MDR microorganisms. This study was designed to use the culture supernatant of *Massilia* sp. MAHUQ-52 for the facile, non-toxic and eco-friendly synthesis of AgNPs, which were evaluated for their acceptability as an antimicrobial agent to control the multi-drug resistant pathogenic *K. pneumoniae* and *S.* Enteritidis. This is the first report for the biosynthesis of novel AgNPs using *Massilia* sp. MAHUQ-52 and their antimicrobial efficacy against pathogenic *K. pneumoniae* and *S.* Enteritidis.

## 2. Results and Discussion

### 2.1. Identification and Characterization of AgNPs-Producing Strain MAHUQ-52

**Figure 1:**
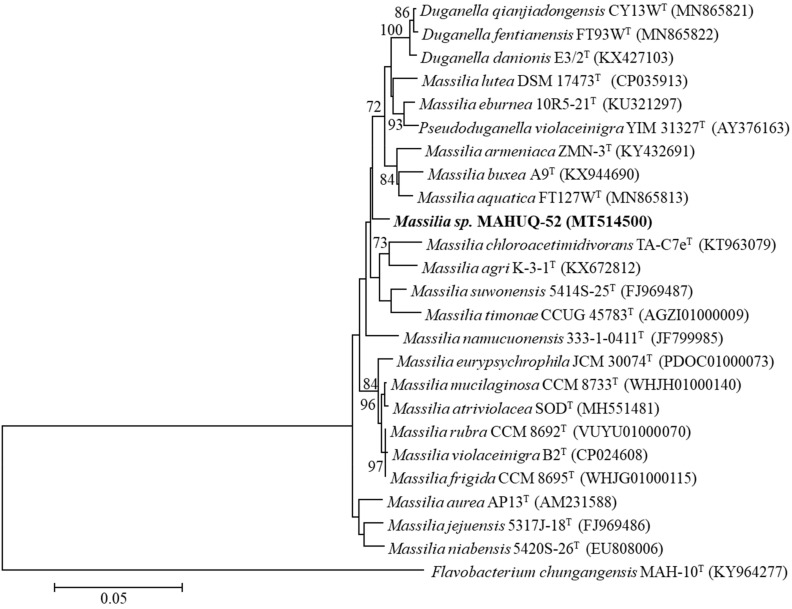
The neighbor-joining (NJ) tree based on 16S rRNA gene sequence analysis showing phylogenetic relationships of strain MAHUQ-52 and the related type strains. Bootstrap values more than 70% based on 1000 replications are shown at branching points. Scale bar, 0.05 substitutions per nucleotide position.

Strain MAHUQ-52 was isolated from the rhizospheric soil of a banana plant and used for the biologically facile synthesis of AgNPs. The 16S rRNA gene sequence of strain MAHUQ-52 was 1461 bp, and the sequence was deposited in NCBI GenBank under accession number MT514500. According to the 16S rRNA gene sequence analysis using EzBioCloud server, strain MAHUQ-52 showed the highest 16s rRNA sequence similarity with *Massilia aurea* AP13^T^ (97.8%). The phylogenetic analysis based on the neighbor-joining method revealed that strain MAHUQ-52 is grouped within the genus *Massilia* (Figure 1). The closest relative *Massilia aurea* AP13^T^ is reported to be a motile, strictly aerobic, nonspore-forming, Gram-negative and straight rod-shaped bacterium, which belongs to the *Oxalobacteraceae* family [31]. Different species of the genus *Massilia* have been isolated from various environments, including air, rock surface, soil, water, and ice core, etc. [32]. Pending further molecular characterization, we will refer to this strain as *Massilia* sp. MAHUQ-52. Strain MAHUQ-52 grows on R2A agar (best growth medium), NA and TSA, but did not grow on LB agar or MacConkey agar. Growth occurs at 10–35 °C (optimum, 30 °C), at pH 6.0–9.0 (optimum, pH 7.0) and with 0–1.0% NaCl (optimum, 0%). Strain MAHUQ-52 has been deposited in KACC (KACC 21999).

### 2.2. Biosynthesis of AgNPs Using Massilia *sp.* MAHUQ-52

**Figure 2:**
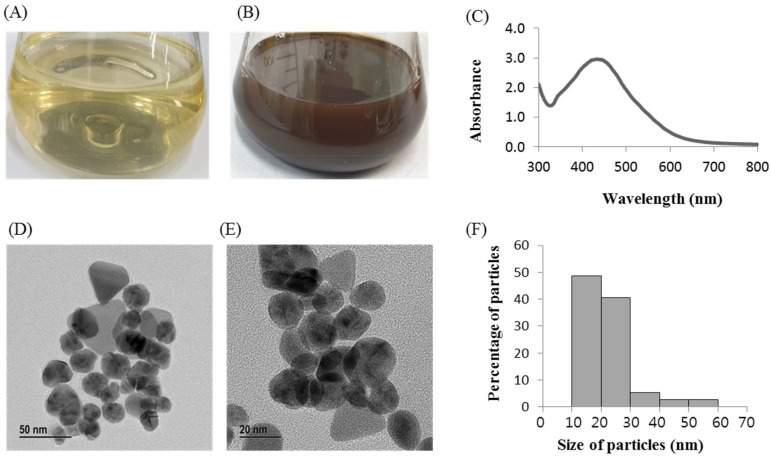
R2A broth with AgNO_3_ as control (**A**), *Massilia* sp. MAHUQ-52 mediated synthesized AgNPs (**B**), UV–vis spectra (**C**), FE-TEM images of synthesized AgNPs (**D**,**E**) and histogram of various sizes of synthesized AgNPs (**F**).

*Massilia* sp. MAHUQ-52 reduced the silver ions to silver NPs. The biosynthesis of AgNPs was confirmed by changing from light yellow color to dark brown after 48 h of incubation with 1 mM aqueous AgNO_3_ into the cell-free *Massilia* sp. MAHUQ-52 supernatant (Figure 2B). However, no color change was found in the control incubated under the same conditions without cell-free supernatant (Figure 2A). The formation of dark brown color depends on the surface plasmon resonance. The optimum incubation time (48 h) for green and facile synthesis of AgNPs was examined based on UV-vis spectral analysis (Supplementary Materials Figure S1). There are two methods (extracellular and intracellular) that are available for the biosynthesis of AgNPs using bacteria. Among these two methods, the extracellular method is facile, rapid and easy compared to the intracellular method that requires complex purification steps [33]. In our present study, the facile and convenient extracellular methodology was used to synthesis the AgNPs using *Massilia* sp. MAHUQ-52. Our results agree with Singh et al. [34], who mentioned that when cell-free culture supernatant of *Solibacillus isronensis* sp. was added to AgNO_3_ solution and incubated for 48 h the color of the reaction mixtures changed from pale yellow to dark brown. Rhizosphere bacteria play vital roles in biotransformation, phytoremediation, plant nutrition, growth promotion, etc. [22,28]. Therefore, the rhizosphere bacterial strain *Massilia* sp. MAHUQ-52 was considered the most potent isolate for the biosynthesis of AgNPs due to the color changes and maximum absorption peak.

### 2.3. Characterization of Biosynthesized AgNPs

**Figure 3:**
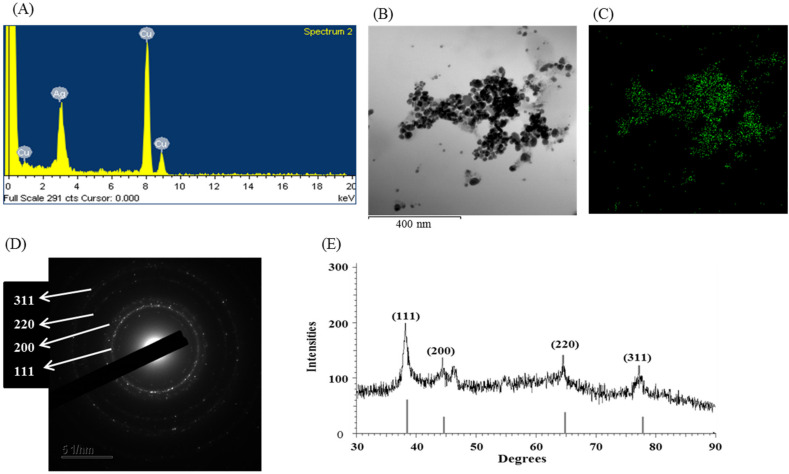
EDX spectrum of synthesized AgNPs (**A**), TEM image used for mapping (**B**), distribution of silver in elemental mapping (**C**), SAED pattern (**D**) and X-ray diffraction pattern (**E**) of *Massilia* sp. MAHUQ-52 mediated synthesized AgNPs.

The synthesis of AgNPs using *Massilia* sp. MAHUQ-52 was confirmed by UV-visible spectrophotometry within 300–800 nm range which showed an absorption peak of 435 nm (Figure 2C), indicating the existence of AgNPs. Depending on nanoparticle (NP) properties, such as shape, size and capping agents, the exact location of the SPR band can vary [35]. According to Brause et al., the absorption pick is associated with NP size [36]. The SPR of different metal NPs in an aqueous solution increases to longer wavelengths by increasing the particle size. Krishnaraj et al. reported that the position and form of metal NP absorption pick are strongly dependent on the size of the particle, stabilizing molecules and the bioelectricity of media [37]. Our results agree with Akter et al. [26], who mentioned that the silver SPR band of *Terrabacter humi* mediated synthesized NPs occurred at 413 nm. The morphology (shape and size) of synthesized NPs were analyzed by FE-TEM. The TEM analysis revealed the spherical shape of biosynthesized AgNPs (Figure 2D,E). The size of *Massilia* sp. MAHUQ-52 mediated synthesized AgNPs was in a range of 15–55 nm. Figure 2F showed the histogram of sizes of green synthesized AgNPs. The average size of green synthesized AgNPs was 23.2 nm. A similar particle size was formed by *Cedecea* sp. in the range of 10–40 nm [38]. Energy dispersive X-Ray (EDX) and elementary mapping was used to investigate the purity and elemental composition of synthesized AgNPs. In the present study, EDX spectroscopy analysis was carried out for AgNPs produced by *Massilia* sp. MAHUQ-52 (Figure 3A–C), which ensured the presence of elemental silver based on the signals. In the EDX spectrum, the synthesized NPs showed a peak at 3 keV, which was due to the presence of silver nanocrystallites [26]. Two other peaks were also found due to the presence of copper grids (Figure 3A). The elemental mapping results of the synthesized nanoproducts reveal the maximum distribution of silver elements, suggesting that silver was the predominant element in the synthesized NPs (Figure 3B,C; Table 1).

The XRD pattern of synthesized NPs revealed intensive peaks throughout the two-party scope in the range of 30–90°, similar to the Bragg’s AgNPs reflection, which indicated the formation of AgNPs. AgNPs produced by *Massilia* sp. MAHUQ-52 showed distinguished XRD peaks at 38.25, 44.65, 64.90 and 77.80° with 2θ values (Figure 3E). These peaks are corresponding to the 111, 200, 220 and 311 reflection planes of face-centered-cubic (fcc) silver, respectively. Our findings agree with Du et al. [23] and Singh et al. [27], who showed a similar XRD pattern of AgNPs synthesized by bacteria. SAED analysis showed sharp rings which indicate the crystalline nature of AgNPs and corresponding to the reflections of 111, 200, 220, and 311 (Figure 3D). Similar results were reported by Huq [4]. The DLS analysis revealed that the average particle size of the synthesized AgNPs was around 109.3 nm, and the polydispersity index was 0.294 (Supplementary Figure S2). The zeta potential value of the aqueous AgNPs solution was −18.4 mV (Supplementary Figure S3). The size of biosynthesized AgNPs is different from the results found from FE-TEM analyses. This may be because DLS measures the total hydrodynamic radius of the AgNPs in solution, which includes conjugated molecules instead of particle size.

**Figure 4:**
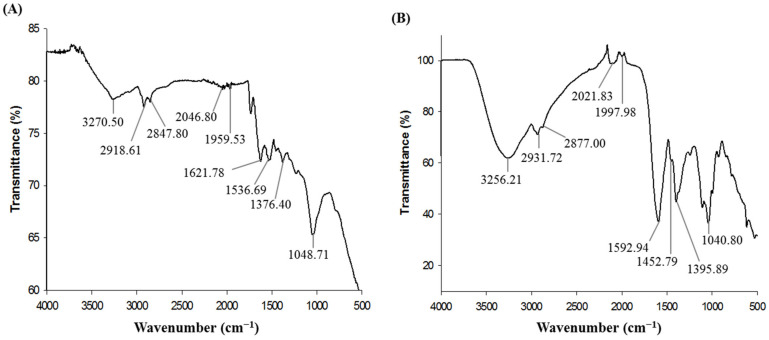
FT-IR spectra of *Massilia* sp. MAHUQ-52 mediated synthesized AgNPs (**A**) and bacterial culture supernatant (**B**).

Fourier-transform infrared (FT-IR) analysis was performed to characterize the surface chemistry and identify the functional molecules involved with the biosynthesis of AgNPs by *Massilia* sp. MAHUQ-52. FT-IR spectrum of air-dried powder of purified AgNPs displayed the vibrational stretches at 3270.50, 2918.61, 2847.80, 2046.80, 1959.53, 1621.78, 1536.69, 1376.40 and 1048.71 cm^−1^ (Figure 4A). The vibration band at 3270.50 cm^−1^ can be attributed to stretching vibrations of O-H (alcohol) and/or N-H (amine). The spectral peaks at 2918.61 and 2847.80 cm^−1^ correspond to the C-H group. The absorbance peaks at 2046.80 and 1959.53 cm^−1^ can be represented as alkyne group. Peaks found at 1621.78 and 1536.69 cm^−1^ can be ascribed to C=O (ester) or -C=C bond. The fingerprint region showed spectral peaks at 1376.40 and 1048.71 cm^−1^ can be attributed to C-H bending or COO- groups and C-O (alcohol/ether) or C-N (amine) stretching, respectively. FT-IR spectrum of bacterial culture supernatant also displayed the identical vibrational stretches at 3256.21, 2931.72, 2877.00, 2021.83, 1997.98, 1592.94, 1452.79, 1395.89 and 1040.80 cm^−1^ (Figure 4B). The results of this study coincided with other reports [23,27,38]. FT-IR analysis revealed the binding of carbohydrates, proteins, and phospholipids with synthesized NPs which contributed for the stability of NPs, as well as capping and functionalizing agents.

### 2.4. Antimicrobial Activity of Biosynthesized AgNPs

**Figure 5:**
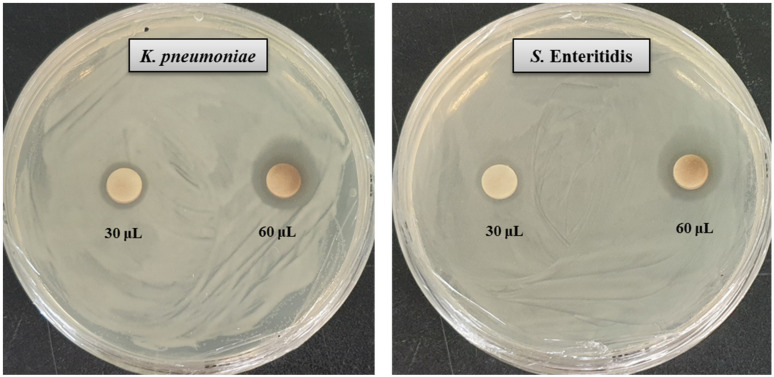
Zones of inhibition of biosynthesized AgNPs (30 μL and 60 μL at 1000 ppm concentrations in water) against *K. pneumoniae* and *S.* Enteritidis.

**Figure 6:**
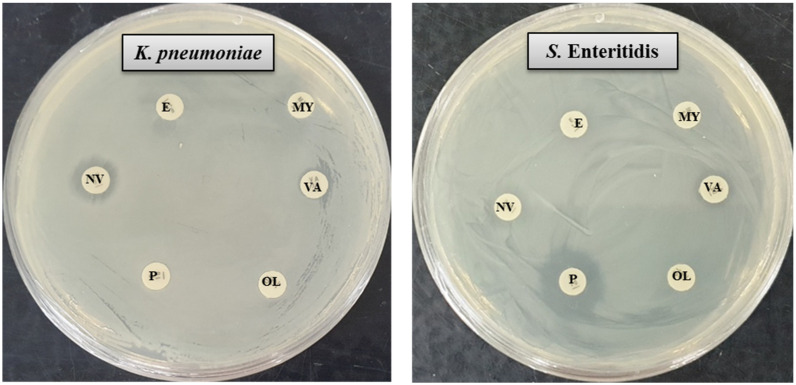
Zones of inhibition of commercial antibiotics against *K. pneumoniae* and *S.* Enteritidis. Abbreviations: E (erythromycin, 15 μg/disc), VA (vancomycin, 30 μg/disc), MY (lincomycin, 15 μg/disc), OL (oleandomycin, 15 μg/disc), P (penicillin G, 10 μg/disc), and NV (novobiocin, 30 μg/disc).

NPs have a high surface–volume ratio with a small size that makes them capable to interact with microbial surfaces. The large surface area of AgNPs promotes their interaction with pathogens to perform antimicrobial activities [38]. In the present study, the inhibitory potential of *Massilia* sp. MAHUQ-52 mediated synthesized AgNPs against two multi-drug resistant human pathogenic bacteria *K. pneumoniae* and *S.* Enteritidis was investigated by disk diffusion assay. The potential antimicrobial properties of biosynthesized AgNPs against tested pathogens were confirmed by the formation of a clear zone of inhibition (ZOI) (Figure 5). The formation of clear ZOI ensured the complete growth inhibition of *K. pneumoniae* and *S.* Enteritidis. The diameters of ZOI are shown in Table 2. The diameters of inhibition zone of *Massilia* sp. MAHUQ-52 mediated synthesized AgNPs *against K. pneumoniae* and *S.* Enteritidis were 17.6 ± 0.5 and 16.8 ± 0.9 mm, respectively. These findings are consistent with previous studies that showed the antimicrobial activities of biosynthesized AgNPs against *Bacillus cereus* and *Pseudomonas aeruginosa* [17]. Six standard antibiotic disks, including erythromycin, vancomycin, penicillin, novobiocin, oleandomycin and lincomycin, were also used as a control to check their antibacterial efficacy. It was found that *S.* Enteritidis showed a resistant pattern against five tested antibiotics. From Figure 6, it was found that only penicillin had antibacterial efficacy against *S.* Enteritidis (Table 2). Conversely, only novobiocin showed activity against *K. pneumoniae*, but the other five antibiotics—erythromycin, vancomycin, penicillin, oleandomycin and lincomycin—did not show any activity against *K. pneumoniae* (Figure 6, Table 2). The inhibition potential of *Massilia* sp. MAHUQ-52 mediated synthesized AgNPs against *K. pneumoniae* and *S.* Enteritidis was significantly higher than the tested antibiotics. The inhibitory action of AgNPs against pathogens due to their small size and large surface area make it perfect to interact with the microbial cell membrane [38]. *Massilia* sp. MAHUQ-52 mediated synthesized AgNPs can be used to overcome the resistance pattern of *K. pneumoniae* and *S.* Enteritidis.

### 2.5. MIC and MBC

**Figure 7:**
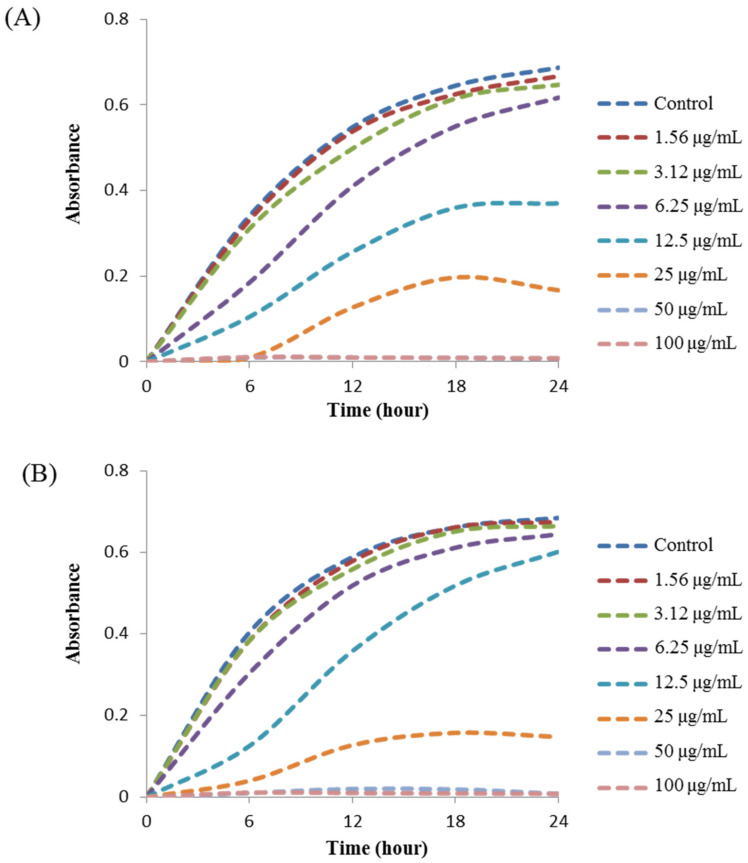
Growth curves of *K. pneumoniae* (**A**) and *S.* Enteritidis (**B**) cultured in MHB with different concentrations of *Massilia* sp. MAHUQ-52 mediated synthesized AgNPs to determine MIC.

**Figure 8:**
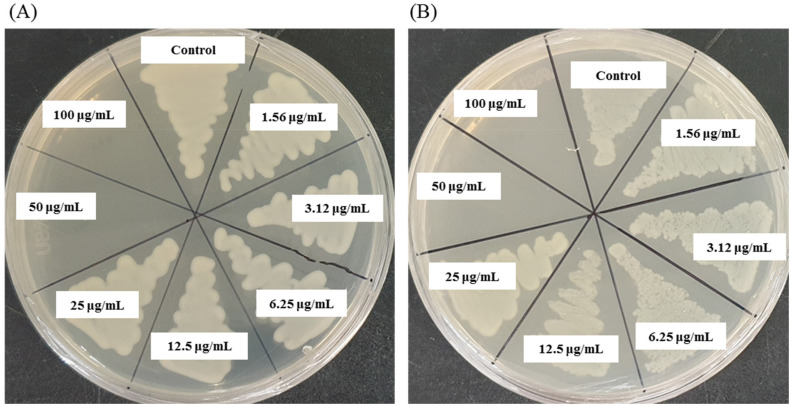
MBC of *Massilia* sp. MAHUQ-52 mediated synthesized AgNPs against *K. pneumoniae* (**A**) and *S.* Enteritidis (**B**).

The MIC values of synthesized AgNPs against *K. pneumoniae* and *S.* Enteritidis were evaluated by a two-fold microdilution assay using 96 well plates. MIC is the lowest concentration of NP that fully inhibits the growth of pathogens. The MICs of *Massilia* sp. MAHUQ-52 mediated synthesized AgNPs for *K. pneumoniae* and *S.* Enteritidis were 12.5 and 25.0 μg/mL, respectively (Figure 7). These MIC values were well below other antimicrobial agents including NPs against *K. pneumoniae* and *S.* Enteritidis. For example, MIC values of biosynthesized zinc oxide, gold and silver NPs against *K. pneumoniae* were 40, 62.5, and 900 μg/mL, respectively [28,39,40]. Similarly, the MIC value of zinc oxide NPs against *Salmonella* spp. was 80 μg/mL [41]. MBC is the minimum concentration of antimicrobial agents that fully kill pathogens. The MBC of *Massilia* sp. MAHUQ-52 mediated synthesized AgNPs for both *K. pneumoniae* and *S.* Enteritidis was 50.0 μg/mL (Figure 8). This MBC value was also well below other antimicrobial agents including NPs against both *K. pneumoniae* and *S.* Enteritidis [29,41]. 

### 2.6. Morphological Characterization of Pathogens Incubated with AgNPs

**Figure 9:**
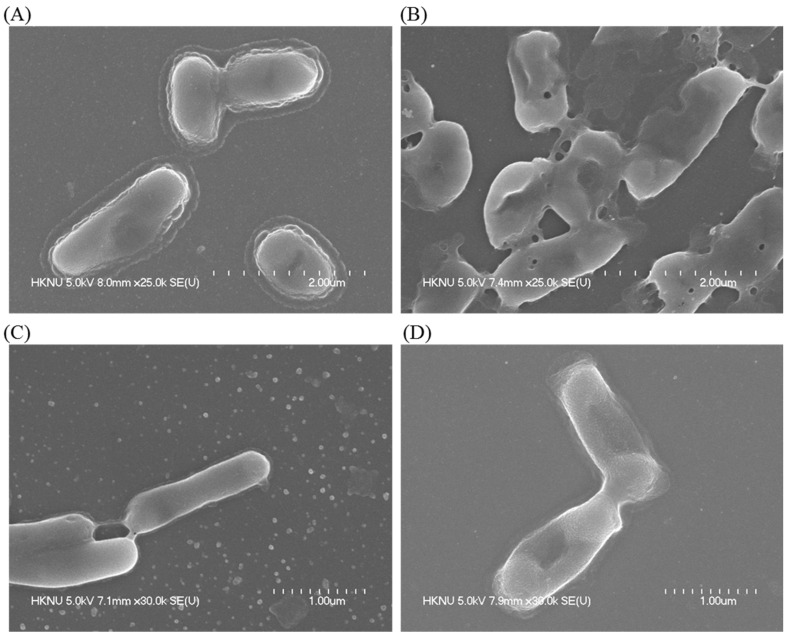
SEM images of normal *K. pneumoniae* cells (**A**), 1×MBC AgNPs treated *K. pneumoniae* cells (**B**), normal *S.* Enteritidis cells (**C**), 1×MBC AgNPs treated *S.* Enteritidis cells (**D**).

FE-SEM analysis was performed to investigate the mechanism of the antibacterial activity of *Massilia* sp. MAHUQ-52 mediated synthesized AgNPs against pathogenic *K. pneumoniae* and *S.* Enteritidis. The nature and degree of the alteration of morphology and damage of the cellular membrane of both pathogens were seen from FE-SEM images (Figure 9A–D). Untreated *K. pneumoniae* had an intact and normal shape (Figure 9A). After incubation with synthesized AgNPs, *K. pneumoniae* showed morphological damage and distortion of the cell wall (Figure 9B). A similar result was found with *S.* Enteritidis. Untreated *S.* Enteritidis had an intact and normal rod shape (Figure 9C). However, after incubation with synthesized AgNPs, *S.* Enteritidis showed morphological changes with damaged, irregular, abnormal cell walls (Figure 9D). This can be attributed to the oxidative stress due to the formation of reactive oxygen species causing the detachment of the membrane. This ultimately leads to cytoplasm shrinkage and cell wall rupture that both culminate in cell death [42,43,44]. 

## 3. Materials and Methods

### 3.1. Materials

Analytical grade silver nitrate (AgNO_3_), ethanol, glutaraldehyde, MH agar and broth were procured from Sigma-Aldrich (St. Louis, MO, USA). R2A agar and R2A broth were collected from MB-cell (Seoul, South Korea) and standard antibiotic disks were obtained from Oxoid Ltd (Basingstoke, England). The pathogenic strains *K. pneumoniae* ATCC 13883 and *S.* Enteritidis ATCC 13076 were obtained from the American Type Culture Collection (ATCC, University Boulevard, Manassas, VA, USA) which were originally isolated from the lungs of pneumonia patients and the gut, respectively.

### 3.2. Isolation, Identification and Characterization of AgNPs-Producing Strain MAHUQ-52

Based on colony morphology, seven bacterial strains were isolated from rhizospheric soil of a banana plant, located in Dighalgram, Magura, Bangladesh using the serial dilution technique [22]. To check the AgNPs synthesis ability, all isolates were cultured separately in 5 mL R2A broth for 48 h at 30 °C. Then, the culture supernatant was collected and incubated with 1 mM AgNO_3_ solution (final concentration) in a shaking incubator for 48 h at 30 °C. Among all of these isolated strains, only one strain (MAHUQ-52) showed strong AgNPs synthesis ability. Then, the strain (MAHUQ-52) was identified through 16S rRNA gene sequence analysis using bacterial universal primers 27F and 1492R [45]. The 16S rRNA gene sequence of strain MAHUQ-52 was submitted to GeneBank, NCBI. The 16S rRNA gene sequences of close relatives were obtained from the EzBioCloud server [46]. The phylogenetic tree was created using the MEGA6 program [47] with the neighbor-joining algorithm to reveal the phylogenetic position of isolated strain MAHUQ-52 [47]. The optimum growth conditions of strain MAHUQ-52 including media, temperature and pH were examined according to the previous report [22]. The isolated strain MAHUQ-52 was deposited to the Korean Agricultural Culture Collection (KACC).

### 3.3. Extracellular Synthesis of AgNPs

The strain MAHUQ-52 was freshly inoculated in an Erlenmeyer flask containing 100 mL of R2A broth medium and incubated for 30 °C in a shaking incubator for 48 h. The culture was centrifuged at 9000 rpm after the incubation period and supernatants were collected for the biosynthesis of AgNPs. A volume of 100 μL of 1 M AgNO_3_ solution (1 mM final concentration) was added to 100 mL of culture supernatant in 250 mL Erlenmeyer flask. The flask was incubated at 30 °C in a shaking incubator with the dark condition. Primarily, the biosynthesis process was monitored by visual observation through the alteration of color and then confirmed by UV-vis spectral analysis. Finally, the biosynthesized NPs were collected from the reaction mixture by high-speed centrifugation and washed with distilled water. The collected AgNPs were air-dried and used for characterization as well as for antimicrobial application.

### 3.4. Characterization of Biosynthesized of AgNPs

AgNPs were characterized using UV-visible spectroscopy (Optizen POP, Mecasys, Daejeon, South Korea) in the range of 300–800 nm, at regular intervals. For FE-TEM analysis, synthesized AgNPs were dissolved in distilled water, diluted and sonicated. A drop of AgNPs solution was placed on a carbon-coated grid, and water was evaporated. Then, samples were examined (size and shape of AgNPs) with field emission–transmission electron microscopy (FE-TEM) (JEM-2100F, JEOL, Tokyo, Japan) at a voltage acceleration of 200 KV. The energy dispersive X-ray (EDX) and selected area diffraction (SAED) were conducted to evaluate the elementary structure of the sample, purity of biosynthesized AgNPs and metallic nature using the detector attached with FE-TEM. To analyze the crystalline nature of the sample, X-ray diffraction (XRD) analysis was performed over the range of 30 to 90° (2θ), operated at 40 KV with 40 mA. Hydrodynamic diameters, polydispersity index (PDI) and zeta potential value (surface charge) of synthesized AgNPs were studied at 23 °C using a Malvern Zetasizer Nano ZS90 (Malvern Instruments, Worcestershire, UK) according to the previous description [23]. The surface chemistry of the sample to determine the functional groups associated with synthesized AgNPs was investigated using FT-IR spectroscopy over the range of 4000–500 cm^−1^. For FT-IR analysis, air-dried green synthesized AgNPs and freeze-dried culture supernatant in powder form were used.

### 3.5. Antimicrobial Activity of Biosynthesized AgNPs

The antimicrobial activity of the biosynthesized AgNPs against pathogenic *K. pneumoniae* and *S.* Enteritidis was investigated by the disk diffusion method (zone of inhibition, ZOI) [27]. Briefly, the pure colonies of *K. pneumoniae* and *S.* Enteritidis were grown in MHB medium for overnight at 37 °C (180 rpm). A volume of 100 μL of overnight culture fresh pathogens was spread uniformly on the MH agar and sterile paper discs were placed on the media. Then, 30 and 60 μL *Massilia* sp. MAHUQ-52 mediated synthesized AgNPs solution (1 mg/mL, dissolved in distilled water) was poured on the paper discs. Similarly, six standard antibiotics (erythromycin, 15  μg/disc; vancomycin, 30  μg/disc; penicillin G, 10 μg/disc; novobiocin, 30 μg/disc; lincomycin, 15  μg/disc and oleandomycin, 15  μg/disc were used as controls against both *K. pneumoniae* and *S.* Enteritidis. All plates were incubated at 37 °C for 24 h to check the zone of inhibition (ZOI). Upon the end of incubation, the plates were observed for the presence of ZOI surrounding each well, which was calculated and expressed in millimeters (mm).

### 3.6. MIC and MBC

The broth microdilution method was used to determine the MIC of synthesized AgNPs against *K. pneumoniae* and *S.* Enteritidis. Shortly, both pathogenic bacteria were grown overnight in MH broth, and the turbidity was adjusted around 1 × 10^6^ CFU/mL. Then, 100 μL of each pathogenic bacteria was added into 96-well plates and equal volume (100 μL) of *Massilia* sp. MAHUQ-52 mediated synthesized AgNPs solution with various concentration (200, 100, 50, 25, 12.5, 6.2, 3.1 and 1.5 μg/mL) was added. As a control, only MH broth was used instead of AgNPs solution. Then, the 96 well plates were incubated in a shaking incubator for 24 h at 37 °C. Every 3 h of interval, absorbance (600 nm) was taken using an ELISA plate reader (LabTech 4000) (BMG LABTECH, Ortenberg, Germany). The MBC of synthesized AgNPs against *K. pneumoniae* and *S.* Enteritidis was determined by streaking 10 μL of each suspension from 96 well plates and incubating for 24 h at 37 °C. Upon the end of incubation, the minimum concentration of NPs that fully killed the pathogens on MHA plates was recorded as the MBC.

### 3.7. Morphological Characterization of Pathogens Incubated with AgNPs

Bacterial strains of *K. pneumoniae* and *S.* Enteritidis (log-phase cells, approximately 1 × 10^7^ CFU/mL) were each incubated with and without AgNPs (at MBC concentration) for 12 h at 37 °C. After the incubation period, samples were collected from both cultures and processed for FE-SEM analysis according to the previous description [17]. Briefly, the overnight-treated cells were centrifuged at 8000 rpm for 5 min, and the supernatant was poured out. Then, the pellets were washed by PBS (pH 7.0) and fixed at room temperature with 2.5% glutaraldehyde for 4 h. Subsequently, the cells were again washed several times by PBS and serially dehydrated with different concentrations of ethanol (30, 50, 70, 90, 95, and 100%) in 10 min intervals at room temperature. Finally, the dehydrated cells were dried by a desiccator and the samples were coated with gold for morphological and structural evaluation by FE-SEM (JSM-7100F, JEOL, Japan) [48].

## 4. Conclusions

This study is an attempt to control multi-drug resistant pathogenic *K. pneumoniae* and *S.* Enteritidis by the facile and eco-friendly synthesized AgNPs. The convenient extracellular methodology was used for the rapid synthesis of AgNPs using *Massilia* sp. MAHUQ-52. FT-IR analysis confirmed the capping of the AgNPs by the biomolecules of the culture supernatant. MAHUQ-52 mediated synthesized AgNPs exhibited promising antimicrobial activity against both *K. pneumoniae* and *S.* Enteritidis. AgNPs showed ZOI of 17.6 ± 0.5 and 16.8 ± 0.9 mm against *K. pneumoniae* and *S.* Enteritidis, respectively. The MIC values of AgNPs against *K. pneumoniae* and *S.* Enteritidis were 12.5 and 25.0 μg/mL, respectively. The MBC was 50 μg/mL for both pathogens. Moreover, the FE-SEM analysis of AgNPs treated cells showed the morphological changes as damaged, irregular, abnormal cell walls. These changes ultimately lead to cytoplasm shrinkage and cell wall rupture that culminated in cell death. Finally, the isolated strain *Massilia* sp. MAHUQ-52 can be useful for the facile and eco-friendly synthesis of AgNPs and *Massilia* sp. MAHUQ-52 mediated synthesized AgNPs can be utilized as an antimicrobial agent against drug-resistant pathogens to overcome microbial threats.

## Figures and Tables

**Table 1:** The number and percentage of chemical elements present in EDX spectrum of *Massilia* sp. MAHUQ-52 mediated synthesized AgNPs.

Element	Weight%	Atomic%
Cu K	57.40	69.58
Ag L	42.60	30.42
Totals	100.00	100.00

**Table 2:** Antibacterial efficacy of *Massilia* sp. MAHUQ-52 mediated synthesized AgNPs and certain commercial antibiotics against *K. pneumoniae* and *S.* Enteritidis.

Pathogenic Species	Zone of Inhibition (mm)							
	AgNPs (30 μL)	AgNPs (60 μL)	Erythromycin	Vancomycin	Oleandomycin	Lincomycin	Penicillin	Novobiocin
*Klebsiella pneumoniae* [ATCC 13883]	12.0 ± 0.7	17.6 ± 0.5	Resistant	Resistant	Resistant	Resistant	Resistant	9.5 ± 0.6
*Salmonella* Enteritidis [ATCC 13076]	11.5 ± 0.6	16.8 ± 0.9	Resistant	Resistant	Resistant	Resistant	14.5 ± 0.8	Resistant

## Data Availability

Data are available on request from the corresponding authors.

## Supplementary Materials

Figure S1: Effect of incubation time on the green synthesis of AgNPs using *Massilia* sp. MAHUQ-52 was checked on the basis of UV-vis spectral analysis after 24 h, 48 h and 72 h of incubation at 30 °C with 1 mM concentration of AgNO3., Figure S2: Particles size distribution of *Massilia* sp. MAHUQ-52 mediated synthesized AgNPs according to intensity (A), number (B) and volume (C), and Figure S3: Zeta potential distribution showed the negative zeta potential value.

## Sample Availability

Not available.
