# Supplementary material for: Biosynthesis, Characterization and Antibacterial Application of Novel Silver Nanoparticles against Drug Resistant Pathogenic Klebsiella pneumoniae and Salmonella Enteritidis
Source: Molecules. 2021 Oct 2;26(19):5996. doi: 10.3390/molecules26195996 (PMC8512087; doi:10.3390/molecules26195996)
Supplement: Supplementary file 1 [file molecules-26-05996-s001.zip › molecules-1369575-supplementary.pdf]

Supplementary file (MAHUQ-52)

Biosynthesis, Characterization and Antibacterial Application of Novel Silver Nanoparticles against Drug Resistant Pathogenic *Klebsiella pneumoniae* and *Salmonella* Enteritidis.

Md. Amdadul Huq[1*], Shahina Akter[2*]

[1] *Department of Food and Nutrition, College of Biotechnology and Natural Resource, Chung-Ang University, Anseong, Gyeonggi-do, 17546, Republic of Korea.*

[2] *Department of Food Science and Biotechnology, Gachon University, Seongnam, 461-701, Republic of Korea.*

[*] Corresponding author:

Md. Amdadul Huq; E-mail: amdadbge@gmail.com ; amdadbge100@cau.ac.kr; Tel: +82-031-670-4568.

Shahina Akter; E-mail: shahinabristy16@gmail.com

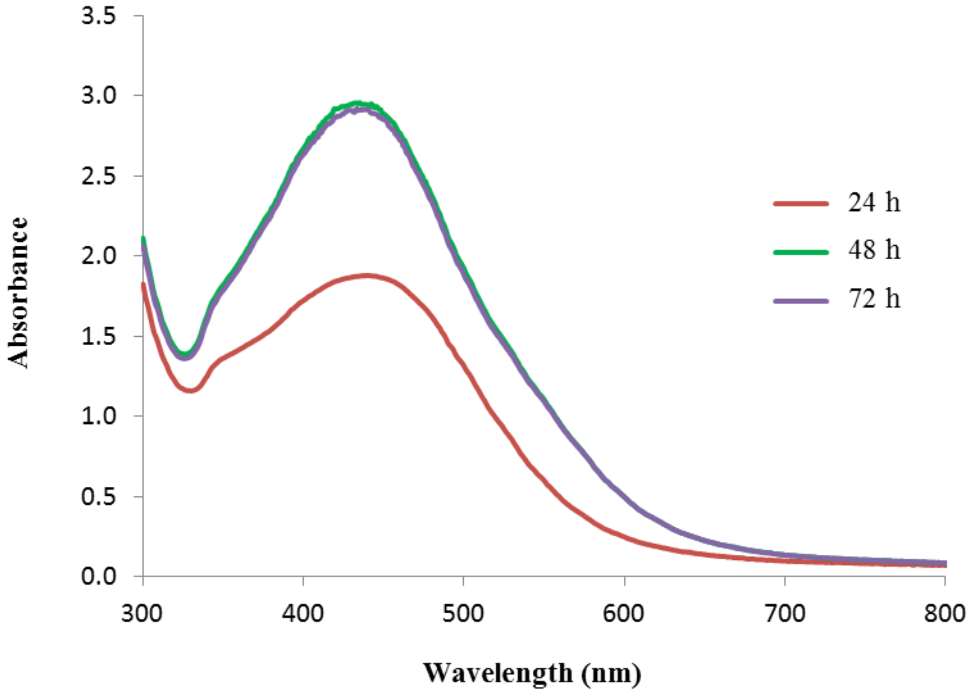

Supplementary Figure S1. Effect of incubation time on the green synthesis of AgNPs using *Massilia* sp. MAHUQ-52 was checked on the basis of UV-vis spectral analysis after 24 h, 48 h and 72 h of incubation at 30 °C with 1 mM concentration of AgNO3.

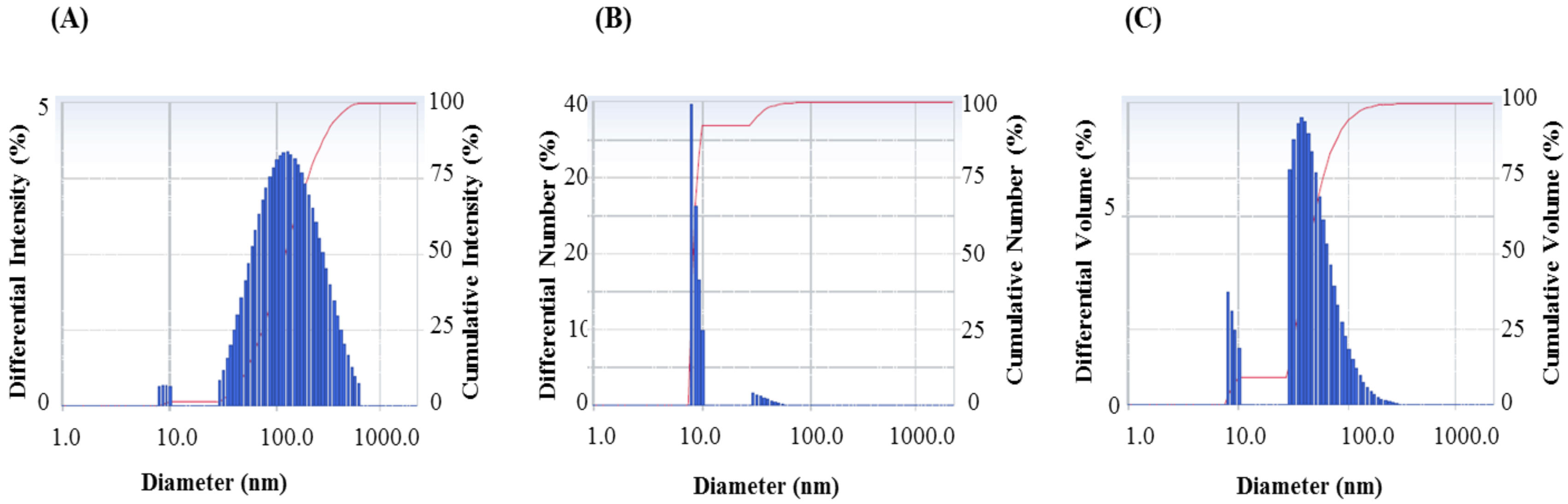

Supplementary Figure S2. Particles size distribution of *Massilia sp.* MAHUQ-52 mediated synthesized AgNPs according to intensity (A), number (B) and volume (C).

Measurement Results

Cumulants Results

Diameter	(d)	: 109.3	(nm)
Polydispersity Index	(P.I.)	: 0.294	
Diffusion Const.	(D)	: 4.499e-008	(cm²/sec)
Residual		: 2.866e-003	(O.K)

Measurement Condition

Temperature	:	25.0	(°C)
Diluent Name	:	WATER	
Refractive Index	:	1.3328	
Viscosity	:	0.8878	(cP)
Scattering Intensity	:	25866	(cps)
Attenuator 1	:	1.95	(%)

Distribution Results (Contin)

Intensity Distribution			Volume Distribution			Number Distribution		
Peak	Diameter (nm)	Std. Dev.	Peak	Diameter (nm)	Std. Dev.	Peak	Diameter (nm)	Std. Dev.
1	9.2	0.8	1	9.0	0.8	1	8.8	0.7
2	166.4	114.4	2	57.8	31.8	2	40.0	11.4
3	0.0	0.0	3	0.0	0.0	3	0.0	0.0
4	0.0	0.0	4	0.0	0.0	4	0.0	0.0
5	0.0	0.0	5	0.0	0.0	5	0.0	0.0
Average	164.3	115.0	Average	53.4	33.3	Average	11.2	9.0

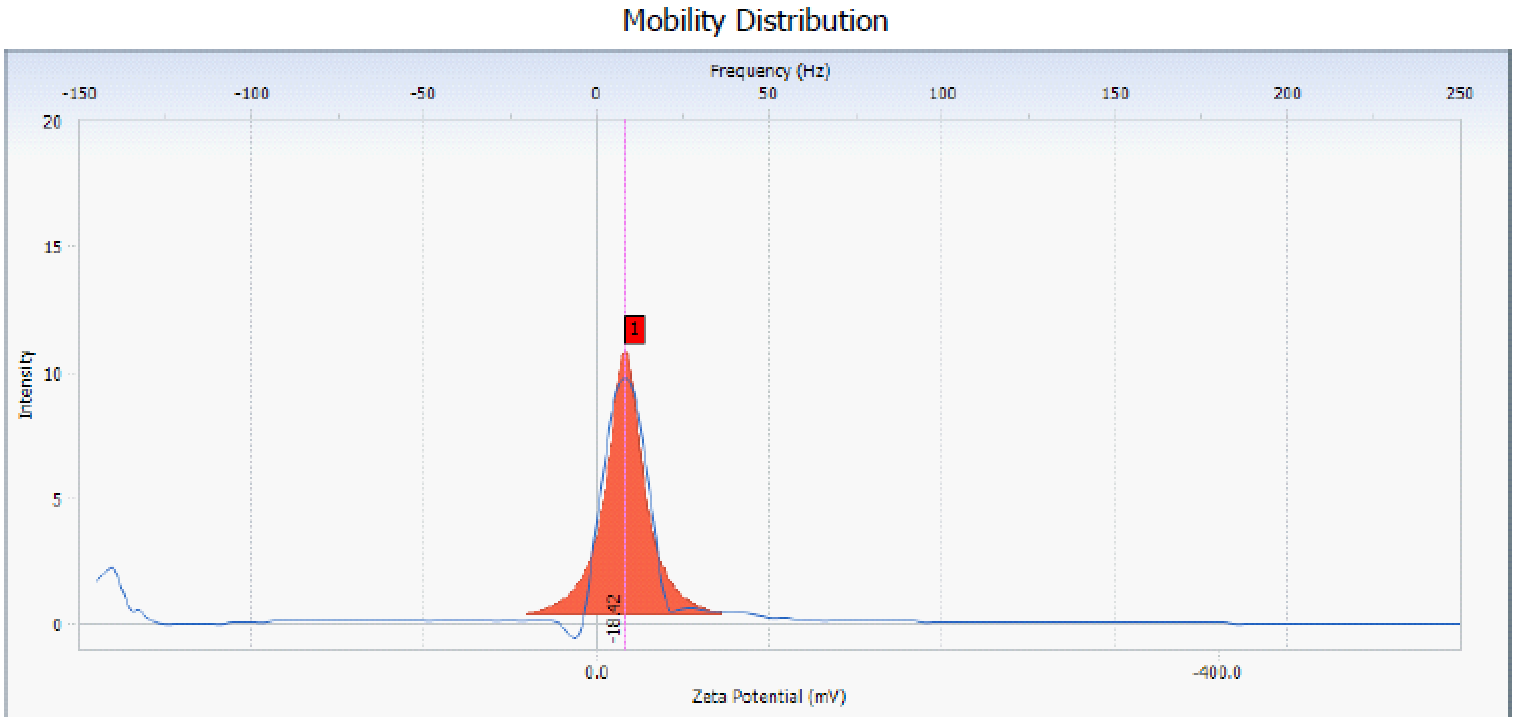

Supplementary Figure S3. Zeta potential distribution showed the negative zeta potential value.

Measurement Results

Zeta Potential	: -18.42	(mV)	Doppler shift	:	8.30	(Hz)
Mobility	: -1.436e-004	(cm²/Vs)	Base Frequency	:	126.5	(Hz)
Conductivity	: 0.0691	(mS/cm)	Conversion Equation	:	Smoluchowski	
Zeta Potential of Cell			Diluent Properties			
Upper Surface	: -80.08	(mV)	Diluent Name	:	WATER	
Lower Surface	: -33.85	(mV)	Temperature	:	25.0	(°C)
Cell Condition			Refractive Index	:	1.3328	
Cell Type	: Zeta Cell (Dispo)		Viscosity	:	0.8878	(cP)
Avg. Electric Field	: -12.02	(V/cm)	Dielectric Constant	:	78.3	
Avg. Current	: -0.04	(mA)				
